# Fracture of a Sivash-Range of Motion Femoral Stem at the Diaphyseal Spline

**DOI:** 10.7759/cureus.20660

**Published:** 2021-12-24

**Authors:** Tyler J Humphrey, Daniel Marchwiany, Hany S Bedair, Christopher M Melnic

**Affiliations:** 1 Orthopaedics, Massachusetts General Hospital, Harvard Medical School, Boston, USA

**Keywords:** stem fracture, total joint arthroplasty, femoral stem design, revision hip and knee replacement, revision hip and knee surgery

## Abstract

We discuss one of the four reported cases involving the fracture of a spline of the Sivash-range of motion (S-ROM) femoral prosthesis. It occurred in a 71-year-old female patient and was fully discovered during stem extraction in revision total hip arthroplasty (THA). The fractured spline was successfully removed using a reverse curette and fluoroscopic guidance. The placement of a new S-ROM femoral prosthesis was successful, and there were no other orthopedic complications.

## Introduction

Introduced in 1984, the Sivash-range of motion (S-ROM) femoral stem (DePuy Synthes, Warsaw, IN), has been used extensively for over 27 years in both revision and primary total hip arthroplasty (THA); its use has been validated by the low documented rates of complications such as dissociation, aseptic loosening, osteolysis, and prosthetic fracture [1]. Modular femoral stems come with many advantages in complex arthroplasty cases, especially the ability to accommodate for proximal-distal femoral bone mismatch, abnormal femoral version, leg-length discrepancy, and the varying offset [2]. However, despite the S-ROM stem’s success, complications, i.e., prosthetic fractures, have been documented in rare instances. Our review of the literature revealed three cases of S-ROM prosthetic fractures distal to the metaphyseal sleeve. In all of these cases, the fracture occurred at least four years following either primary or revision THA [3,4]. The first patient was a 75-year-old female who developed spontaneous atraumatic thigh pain, including pain with weight-bearing, four years after the insertion of an S-ROM stem for primary THA. Another case involved a 68-year-old female who reported atraumatic thigh pain and pain with weight-bearing four years after the insertion of an S-ROM prosthesis for single-stage revision for infection. These two patients were managed with protected weight-bearing with a cane and non-steroidal anti-inflammatory drugs (NSAIDs) and showed no pain or functional deficits after eight weeks of conservative management [3]. The third patient was a 66-year-old female who was asymptomatic and whose routine radiographs of the right hip showed a fracture of the posterior spline of her S-ROM prosthesis, seven years after undergoing primary THA [4]. We present the fourth reported case of a fracture of a spline of an S-ROM prosthesis, which was fully discovered during extraction of the pre-existing S-ROM stem in revision surgery for recurrent hip instability and dislocation.

Informed consent was obtained from the patient for the use of her case information for publication, including deidentified case details, health history, radiographs, and intraoperative photographs.

## Case presentation

**Figure 1:**
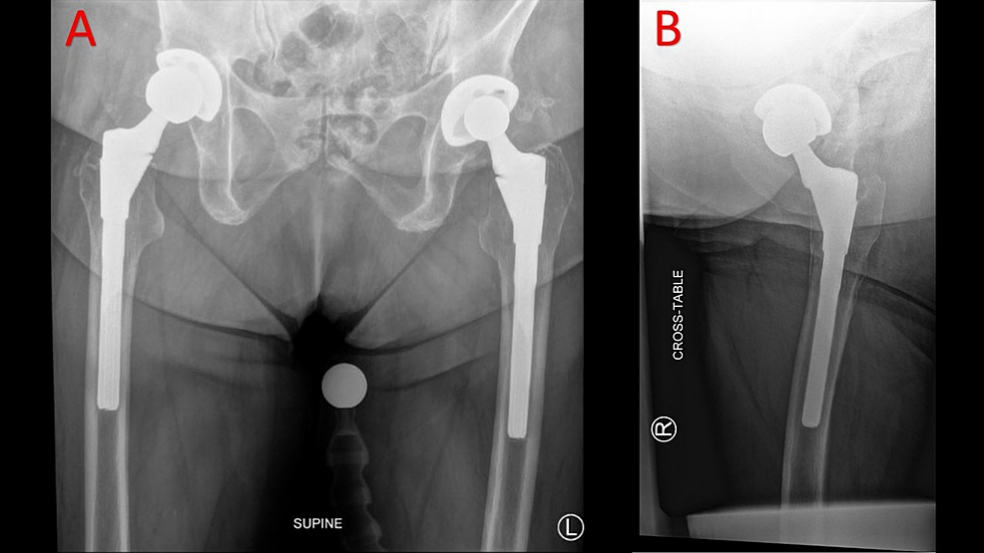
Preoperative radiographs Panel A: Preoperative anterior-posterior pelvis radiograph demonstrating minimal anteversion of the right acetabular component. Panel B: Preoperative cross-table lateral radiograph of the right hip

**Figure 2:**
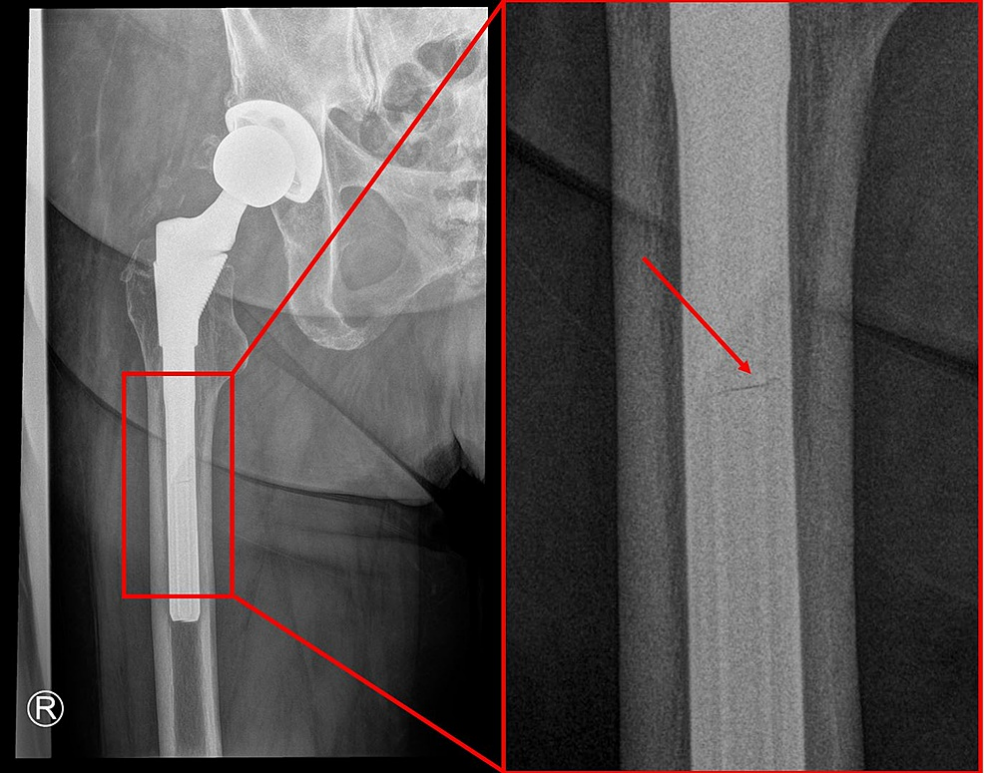
Magnified view of the preoperative anterior-posterior pelvic radiograph The magnified anterior-posterior pelvic radiograph appeared to demonstrate a small linear radiolucency thought to represent a partial fracture of the S-ROM diaphyseal stem S-ROM: Sivash-range of motion

The patient was a 71-year-old female with heart failure with preserved ejection fraction (HFpEF), hypertension, diabetes mellitus type II, obstructive sleep apnea, lumbar fusion in 2012, and obesity (BMI of 39 Kg/m^2^) with a history of right THA with S-ROM placement in 1998 with subsequent headliner exchange for polyethylene wear and instability in 2018. The component details of the S-ROM prosthesis as of 2018 were as follows: 18 x 13 x 160-mm S-ROM prosthesis with +9-mm neck offset. Following this revision surgery in 2018, the patient was pain-free, able to fully ambulate, and had no issues performing activities of daily living. However, in 2020, the patient sustained two subsequent low-energy dislocations. The patient first presented to our clinic following these dislocations and was pain-free at the time. Her preoperative radiographs initially demonstrated a well-fixed right total hip replacement with minimal anteversion of the acetabular component appreciated on the cross-table lateral radiograph (Figure 1). Upon further examination, the patient's anterior-posterior pelvis radiograph appeared to demonstrate a small linear radiolucency in the diaphyseal stem, thought to represent a partial fracture of the prosthesis (Figure 2).

Although the patient was pain-free in the setting of a possible partial S-ROM diaphyseal stem fracture, we offered her a re-revision of the hip arthroplasty with a plan to revise one or both components due to her recurrent instability and concern for an under-anteverted acetabular component. Additional preoperative workup for serum erythrocyte sedimentation rate (ESR) and C-reactive protein (CRP) to rule out infection revealed levels of 15 mm/h and <3.0 mg/L, respectively. Preoperative serum cobalt and chromium levels to evaluate for trunnionosis were each <1.0 ng/mL. Preoperative Patient-Reported Outcome Measure (PROM) values of the Hip disability and Osteoarthritis Outcome Score (HOOS-PS) and Patient-Reported Outcome Measurement Information System (PROMIS) Physical Function Short Form 10a were 62.3 and 39.3, respectively.

Operative procedure

**Figure 3:**
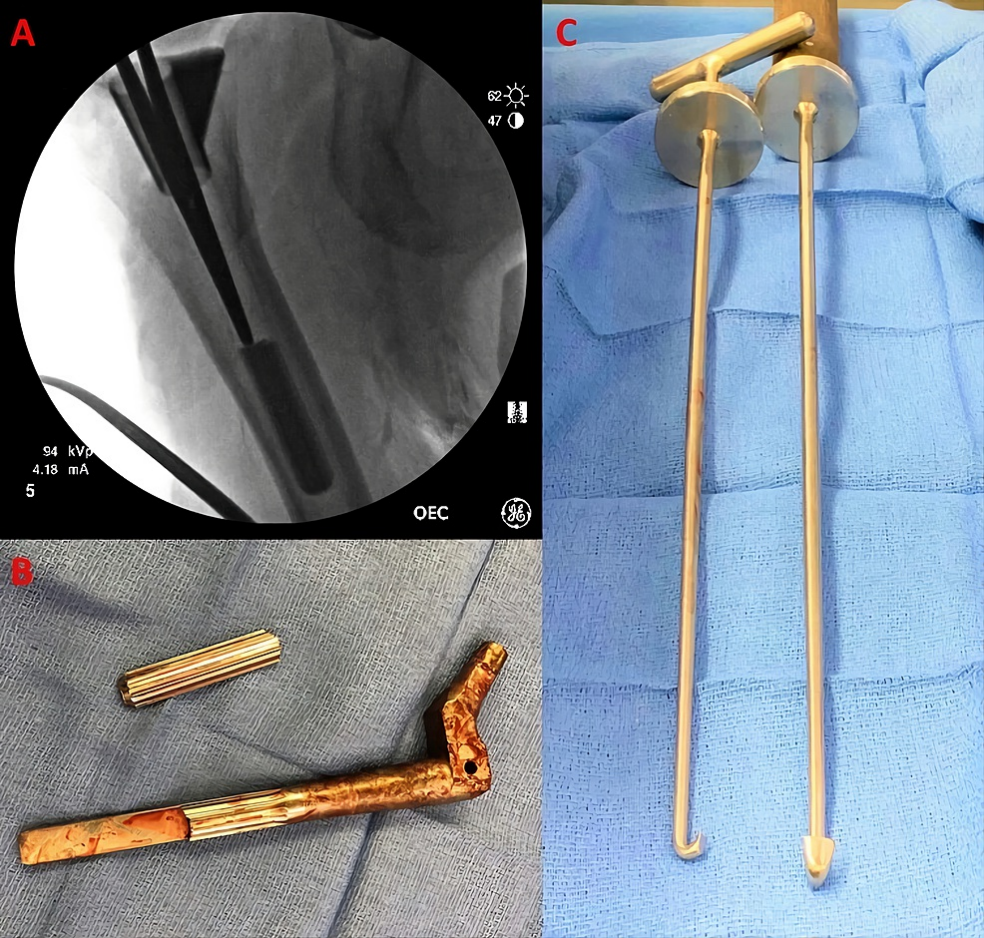
Intraoperative findings and instrumentation Panel A: Intraoperative fluoroscopy demonstrating long forceps adjacent to the fractured posterior spline of original S-ROM prosthesis. Panel B: Intraoperative photograph demonstrating fractured remains of original S-ROM femoral prosthesis after the complete removal from the patient’s femur. Panel C: Full-length photograph of reverse curette used for fractured spline removal S-ROM: Sivash-range of motion

Under general anesthesia, the patient was positioned in lateral decubitus on a radiolucent flattop table. A posterolateral approach was utilized. The hip was dislocated and the trunnion was assessed and noted to be free of corrosion. The stem was found to be in the neutral version; hence, the decision was made to remove the stem and increase the anteversion. A chisel and stacked osteotomes were used to disengage the stem-sleeve taper. We then placed an extractor and vigorously malleted it in an attempt to remove the stem; however, in the process of stem removal, the posterior spline audibly fractured and remained in the femoral canal still well-fixed to the femur. Fluoroscopy was used for visualization and the broken spline was disengaged and removed with a reverse curette and mallet to disengage the spline from the posterior cortex (Figure 3). The sleeve was well-fixed and retained.

The acetabular component was found to be well-fixed and with only minimal anteversion. Following cup removal, there was bone loss in the anterosuperior column, which was consistent with a Paprosky 2A defect [5].

**Figure 4:**
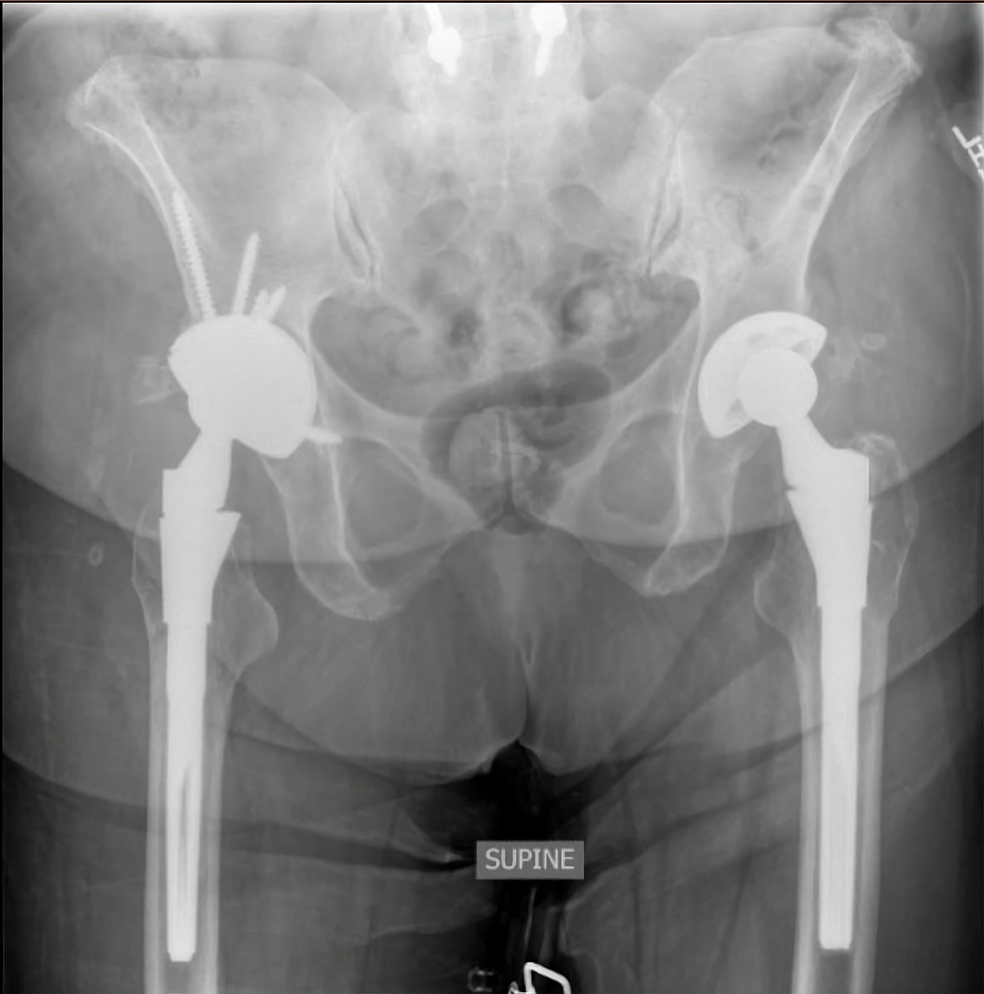
Immediate postoperative anterior-posterior pelvis radiograph This radiograph demonstrates the new S-ROM femoral prosthesis and a 58-mm multi-hole acetabular cup with 20 degrees of added anteversion. Seven screws were placed, with two of these screws being ischial S-ROM: Sivash-range of motion

We were able to ream medially and obtained good column fixation with a 58-mm multi-hole cup with 25 degrees of added anteversion. Seven screws were placed, with two of the screws being ischial (Figure 4). A trial dual-mobility liner and trial stem were placed with 20 degrees of added anteversion. The hip was trialed and was found to be stable.

The dual mobility liner was then inserted and assessed to be fully seated. The final stem was inserted with 20 degrees of anteversion (S-ROM 42 standard neck, 18 x 1 x 160 mm). With final implants, the hip was assessed and found to be stable. Postoperatively, the patient was immediately made toe-touch weight-bearing, with global (anterior and posterior) hip dislocation precautions and an abduction brace.

Outcome and follow-up

**Figure 5:**
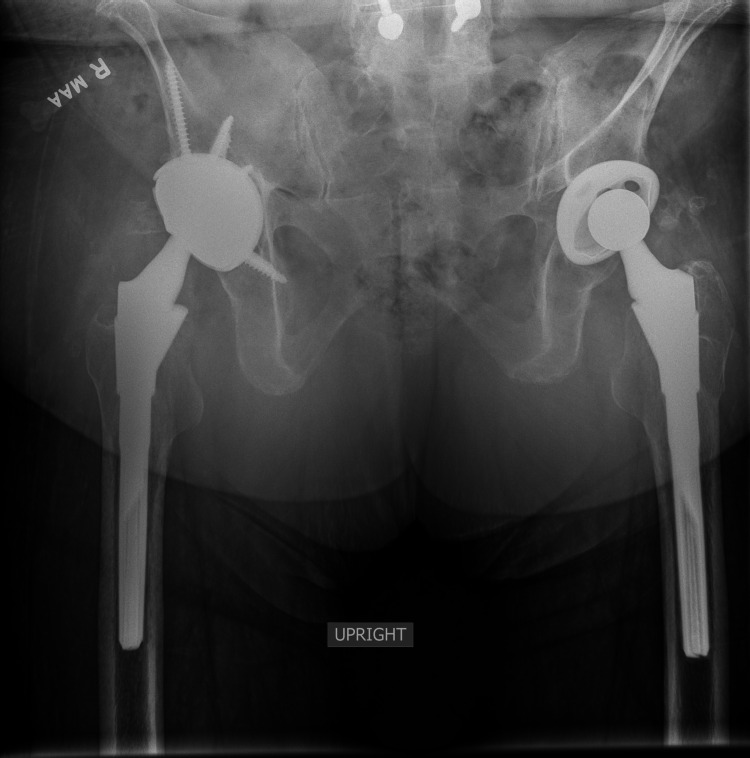
Three-month postoperative anterior-posterior pelvis radiograph The radiograph shows well-seated and aligned implants with no evidence of loosening or other component complications

There were no acute surgical complications. The patient was discharged to an inpatient rehabilitation facility for one week before she returned home. At the one-month follow-up, the patient was found to be doing well. She had advanced to 50% weight-bearing status at the one-month visit and ultimately advanced to weight-bearing as tolerated at the three-month follow-up appointment given her pain-free progression of mobility. At both the one-month and three-month follow-up visits, radiographs of the right hip showed well-seated and aligned implants with no evidence of loosening or other component complications (Figure 5). Three-month postoperative HOOS-PS and PROMIS Physical Function Short Form 10a scores were 49.2 and 31.8, respectively, and the patient’s reported daily-average pain levels were 1/10 (with 10 representing the worst possible pain).

## Discussion

The S-ROM hip system’s reliability and excellent long-term results have made it a useful option in both primary and revision hip arthroplasty. In this case report, we discuss one of four reported cases of the fracture of a spline of the S-ROM prosthesis, which was fully discovered during stem extraction. As there are only 18 known reports (including the present study) of fractures of the S-ROM prosthesis at different prosthetic locations, and at different time points postoperatively [3,4,6-12], it is important to document trends in implant survivorship (Table 1).

**Table 1:** Reported S-ROM prosthesis fractures in the literature *Based on authors' suspicion. **Seven cases were presented. Certain variables are presented as averages, as this study was not a case series with patient-level information available. All patients had revision THA as management BMI: body mass index; S-ROM: Sivash-range of motion; THA: total hip arthroplasty ZT and ZTT are trademarked names for the S-ROM stem from DePuy Synthes

Authors and year	Patient age at diagnosis (years), gender	Patient BMI (kg/m^2^)	Available stem details	Likely reason for failure*	Location on S-ROM stem	Years till failure	Management
Current case	71, female	39	18 × 13 × 160-mm S-ROM prosthesis with +9-mm neck offset	Acute bending forces during extraction, fatigue	Diaphyseal stem	23	Completion of revision THA
Pearce et al., 2014 [3]	71, female	N/A	16 × 11 × 150-mm with a 36 + 6-mm lateral offset neck, 16F XXL ZT^TM^ hydroxyapatite-coated proximal sleeve	Fatigue	Diaphyseal stem	4	Conservative
Pearce et al., 2014 [3]	68, female	N/A	16 × 11 × 150-mm with a 36 + 6-mm lateral offset neck, hydroxyapatite-coated proximal sleeve	Fatigue	Diaphyseal stem	4	Conservative
McNabb et al., 2016 [4]	66, female	22	16 × 11 × 150-mm with a 36 + 6-mm proximal geometry, with a 16B large ZTT^TM^ metaphyseal sleeve	Fatigue	Diaphyseal Stem	7	Conservative
Rueckl et al., 2017 [6]	52, female	31	14 × 9 × 130-mm stem, 36-mm standard neck, 14D large proximal sleeve	Fatigue	Stem-sleeve interface	3	Revision THA
Parisi et al., 2015 [7]	50, N/A	32.5	16 × 11 × 150-mm with a 36 + 6-mm neck, 16B large ZTT^TM^ sleeve	Fatigue	Stem-sleeve interface	7	Revision THA
Mehran et al., 2013 [8]	61, male	N/A	3618L S-ROM stem, 18 × 13 × 160-mm, and an 18F large ZTT^TM^ sleeve	Fatigue	Stem-sleeve interface	9	Revision THA
Waly et al., 2015 [9]	64, female	28	14 × 9-mm stem, 36 standard neck, 14B sleeve	Fatigue	Stem-sleeve interface	7	Revision THA
Shah et al., 2017 [10]	77, male	34	13 stem, standard neck	Fatigue	Stem-sleeve interface	10	Revision THA
Patel et al., 2009 [11]	54, male	N/A	S-ROM 18/13, 36 + 8-mm lateral neck, 28-mm ceramic zirconia head, with a 0 Morse taper, and an 18B large ZTT^TM^ sleeve	Fatigue	Stem-sleeve interface	5	Revision THA
Patel et al., 2009 [11]	55, male	N/A	S-ROM 20/15 femoral component, a B large ZT^TM^ sleeve, and a 36 + 8-mm lateral offset neck	Fatigue	Stem-sleeve interface	<1	Revision THA
Huot Carlson et al., 2012** [12]	Average of 66, N/A	Average of 32	11.6-mm stem diameter	Fatigue	Stem-sleeve interface	Average of 9.4	Revision THA

The S-ROM modular femoral prosthesis consists of an independent proximal porous-coated sleeve, a titanium alloy diaphyseal stem with distal flutes, and a coronal slot separating two splines. The distal flutes are designed to enhance rotational stability in the diaphysis without fixation while the coronal slot is designed to reduce distal stem stiffness in the hopes to reduce anterior thigh pain [13].

The point of weakness of modular femoral prostheses, including the S-ROM, has been described at the stem-sleeve junction, head-neck junction, and the modular interface. At these interfaces between modular components, corrosion, metal ion generation, and fretting may occur [14,15]. This corrosion concentrated in the neck/sleeve region can reduce prosthesis integrity, especially when coupled with small-diameter diaphyseal stems and large neck offsets in obese patients, resulting in “fatigue fracture” due to massive bending forces on the neck-sleeve or sleeve-stem interface [6]. Approximately 14 cases of S-ROM fractures at the modular neck due to this “fatigue fracture” mechanism exist in the literature [6-12]. Pearce et al. in 2014 and McNabb et al. in 2016 described the only three known cases of S-ROM spline fracture, which they attributed to the “fatigue fracture” mechanism as well [3,4]. In all cases, the patients were managed expectantly as the proximal porous-coated sleeve maintained excellent bony fixation and stability. Our case is interesting, as our patient initially presented with recurrent dislocations, but was pain-free, similar to the case reported by McNabb et al. [4]. It is plausible that our patient's likely partial S-ROM diaphyseal fracture would have gone unnoticed had it not been for her recurrent dislocations, which prompted an evaluation. Nevertheless, it is plausible that the "fatigue" mechanism, coupled with the forces on the stem from recurrent dislocation, accounted for our patient's preoperative, likely partially fractured S-ROM stem. Interestingly, the patient's fractured S-ROM stem had no gross evidence of osteolysis or corrosion in the sleeve interface region at the time of revision surgery (Figure 3).

Prior studies have demonstrated that removal of an S-ROM prosthesis can be difficult due to cold-welding of the sleeve to the stem, extensive proximal femoral bony fixation (limiting access to the splines), and bony on-growth of the splines themselves [6]. We elected to extract the patient’s S-ROM prosthesis using a chisel and stacked osteotomes to disengage the bony attachments and an extractor with a mallet to remove the stem, a method discussed in a review by Laffosse in 2016 [16]. The complete S-ROM spline fracture occurred at this time of the operation. We believe that a possible preoperative S-ROM spline fracture, coupled with bony on-growth of the splines, and bending forces during extractor malleting, ultimately led to a complete fracture at the most proximal origin of the posterior spline (Figure 3).

Electing to perform the surgery on a radiolucent table proved to be very advantageous (Table 2). It allowed us to utilize fluoroscopy to visualize the broken spline in the femoral diaphysis, to visualize the position of the reverse curette as it hooked around the distal aspect of the spline, and then to use the mallet to disengage and extract it. Without the fluoroscopic assistance, it would have been difficult to know when the reverse curette was hooked around the distal stem or when the spline first started to move, and the technique likely would not have been successful. We describe this rare incident so that arthroplasty surgeons become aware of the rare S-ROM diaphyseal spline fracture and are prepared to successfully remove a fractured spline if they encounter it intraoperatively.

**Table 2:** Technical pearls from our case CRP: C-reactive protein; ESR: erythrocyte sedimentation rate; S-ROM: Sivash-range of motion

Setting	Technical pearls
Prior to operation	Obtain anterior-posterior and cross-table lateral radiographs of the pelvis; obtain serum ESR, CRP, cobalt ions, and chromium ions
Operative setup	Operating room setup should include a radiolucent table, intraoperative fluoroscopy, reverse curette, chisel, osteotomes, specific stem extraction system (if available), and extraction mallets
Moment of extraction	A chisel and stacked osteotomes may be utilized to disengage the stem-sleeve interface, if a fracture of the distal S-ROM stem is encountered intraoperatively – removal of the remaining stem can be achieved using a reverse curette and extraction malleting to engage the stem piece and retrieve it under fluoroscopic guidance

## Conclusions

We discussed one of the four reported cases of a fracture of a diaphyseal spline of the S-ROM femoral prosthesis. This complication likely occurred due to a preoperative partial “fatigue fracture” of the spline, bony on-growth of the spline, and bending forces on the proximal origin of the spline during extraction malleting. Surgeons should be aware of this exceedingly rare complication of the S-ROM diaphyseal spline and the method used to manage the removal of a fractured spline if it is encountered intraoperatively.
